# A Text-Book of Physiology

**Published:** 1889-06

**Authors:** 


*A Text-book of Physiology*. By AUSTIN FLINT, M.D., LL.D. Pp. 872. Fourth Edition, entirely re-written. London: H. K. LEWIS. 1888.

Flint's *Physiology* is a classic well known and much valued on both sides of the Atlantic. This edition has been entirely re-written, historical references have been much curtailed, discussions of unsettled questions have been avoided, and the work seeks to represent, on the authority of a cultivated teacher, the state of physiological science as it is at the present time.

In comparing the work with the best known English ones, those of Landois and Stirling, of Foster and of McKendrick, we should say that it contains less of matters purely experimental than any of these; that it dwells more on established truths; and that, although it makes no special pretence thereto, it has more direct bearings on the practice of our art. Its peculiarities are, in some senses, its chief recommendation. It supplements our English works, presents new points of view, and exhibits, as compared with them, a differently balanced treatment of the whole subject. In another aspect this special value may militate against its favourable acceptance in this country; this is, when the average British student faces the average British examiner.

One feature of the work, which alone is sufficient to display its American origin, is the excellence of the manner in which it has been brought out. In paper, printing, illustration, and general form and handiness, it is far ahead of the ordinary British specimen of book-making.

